# Supplementary material for: Cabbage stem flea beetle’s (Psylliodes chrysocephala L.) susceptibility to pyrethroids and tolerance to thiacloprid in the Czech Republic
Source: PLoS One. 2019 Sep 20;14(9):e0214702. doi: 10.1371/journal.pone.0214702 (PMC6754130; doi:10.1371/journal.pone.0214702)
Supplement: S1 Table — The model was y = ax+b, with a being log transformed, and the lower and upper confidence limits (CL) shown in parentheses. nd–no CL defined. (PDF) [file pone.0214702.s001.pdf]

S1 Table. Probit regression model parameters and fitted doses of active substances of insecticides describing the mortality of CSFB from Prague and Potěhy localities in 2015. The model was y = ax+b, with a being log transformed, and the lower and upper confidence limits (CL) shown in parentheses. nd – no CL defined.

**population**	**active substance**	**N**	**R^2**	***chi*-square**	**LC50 (95% CL)**	**slope ± SE**
Prague	*lambda*-cyhalotrin	181	0.85	27.7	0.0004 (0.0002/0.0006)	2.60 ± 0.49
	tau-fluvalinate	180	0.97	0.0004	0.008 (nd)	7.14 ± 342
	deltamethrin	177	0.80	38.1	0.0006 (0.0004/0.001)	1.90 ± 0.31
	cypermethrin	123	0.74	44.0	0.006 (0.004/0.009)	2.38 ± 0.36
	esfenvalerate	119	0.42	0.0003	0.007 (nd)	15.7 ± 953
	etofenprox	121	0.67	16.6	0.11 (0.08/0.14)	3.65 ± 0.90
	chlorpyrifos	90	0.72	16.2	0.67 (0.55/0.98)	4.44 ± 1.10
	indoxacarb	120	0.33	24.7	0.03 (0.01/0.06)	1.03 ± 0.21
	acetamiprid	90	0.65	20.9	0.03 (0.03/0.04)	5.63 ± 1.23
	thiacloprid	90	0.13	8.37	0.63 (0.32/7.54)	0.98 ± 0.34
Potěhy	*lambda*-cyhalotrin	198	0.92	46.0	0.002 (0.001/0.003)	2.91 ± 0.43
	tau-fluvalinate	184	0.85	51.4	0.02 (0.01/0.03)	2.32 ± 0.32
	deltamethrin	182	0.85	55.6	0.002 (0.002/0.003)	2.06 ± 0.28
	cypermethrin	131	0.68	23.4	0.02 (0.02/0.03)	2.96 ± 0.61
	esfenvalerate	120	0.81	12.7	0.02 (0.01/0.02)	5.82 ± 1.63
	etofenprox	120	0.63	15.1	0.08 (0.07/0.10)	4.50 ± 1.16
	chlorpyrifos	91	1.00	0.0003	0.42 (nd)	38.3 ± 2,336
	indoxacarb	127	0.50	27.1	0.03 (0.02/0.04)	1.96 ± 0.38
	acetamiprid	119	0.79	26.7	0.02 (0.02/0.03)	8.84 ± 1.71
	thiacloprid	120	0.17	14.0	8.10 (2.60/152)	0.64 ± 0.17
