# Supplementary material for: Cabbage stem flea beetle’s (Psylliodes chrysocephala L.) susceptibility to pyrethroids and tolerance to thiacloprid in the Czech Republic
Source: PLoS One. 2019 Sep 20;14(9):e0214702. doi: 10.1371/journal.pone.0214702 (PMC6754130; doi:10.1371/journal.pone.0214702)
Supplement: S2 Table — The model was y = ax+b, with a being log transformed, and the lower and upper confidence limits (CL) shown in parentheses. nd–no CL defined. (PDF) [file pone.0214702.s002.pdf]

S2 Table. Probit regression model parameters and fitted doses of active substances of insecticides describing the mortality of CSFB from Prague and Potěhy localities in 2016. The model was y = ax+b, with a being log transformed, and the lower and upper confidence limits (CL) shown in parentheses. nd – no CL defined.

**population**	**active substance**	**N**	**R^2**	***chi*-square**	**LC50 (95% CL)**	**slope +/- SE**
Prague	deltamethrin	140	0.70	29.9	0.0002 (0.0001/0.0003)	1.89 ± 0.35
	chlorpyrifos	183	0.80	52.5	0.07 (0.05/0.08)	3.57 ± 0.49
	indoxacarb	120	0.37	18.0	0.02 (0.02/0.03)	2.48 ± 0.59
	acetamiprid	170	0.34	25.9	0.002 (0.0005/0.003)	1.24 ± 0.24
	thiacloprid	120	0.21	16.5	7.92 (2.77/89.7)	0.71 ± 0.17
Potěhy	thiacloprid	118	0.17	12.4	14.1 (3.79/686)	0.63 ± 0.18
