# Supplementary material for: Cabbage stem flea beetle’s (Psylliodes chrysocephala L.) susceptibility to pyrethroids and tolerance to thiacloprid in the Czech Republic
Source: PLoS One. 2019 Sep 20;14(9):e0214702. doi: 10.1371/journal.pone.0214702 (PMC6754130; doi:10.1371/journal.pone.0214702)
Supplement: S3 Table — The model was y = ax+b, with a being log transformed, and the lower and upper confidence limits (CL) shown in parentheses. nd–no CL defined. (PDF) [file pone.0214702.s003.pdf]

S3 Table. Probit regression model parameters and fitted doses of active substances of insecticides describing the mortality of CSFB from Prague and Potěhy localities in 2017. The model was y = ax+b, with a being log transformed, and the lower and upper confidence limits (CL) shown in parentheses. nd – no CL defined.

**population**	**active substance**	**N**	**R^2**	***chi*-square**	**LC50 (95% CL)**	**slope +/- SE**
Prague	*lambda*-cyhalotrin	89	0.85	44.2	0.001 (0.0004/0.002)	1.34 ± 0.20
	tau-fluvalinate	90	0.48	31.8	0.09 (0.06/0.13)	1.71 ± 0.30
	chlorpyrifos	90	0.70	0.001	0.03 (nd)	14.7 ± 641
	thiacloprid	118	0.35	22.8	139 (67.8/534)	1.10 ± 0.23
	Biscaya	87	0.28	17.8	0.79 (0.52/1.36)	1.62 ± 0.38
Potěhy	thiacloprid	120	0.54	35.4	67.8 (42.9/127)	1.62 ± 0.27
	Biscaya	180	0.81	39.3	0.67 (0.50/0.89)	2.63 ± 0.42
