# Supplementary material for: Cabbage stem flea beetle’s (Psylliodes chrysocephala L.) susceptibility to pyrethroids and tolerance to thiacloprid in the Czech Republic
Source: PLoS One. 2019 Sep 20;14(9):e0214702. doi: 10.1371/journal.pone.0214702 (PMC6754130; doi:10.1371/journal.pone.0214702)
Supplement: S4 Table — The model was y = ax+b, with a being log transformed, and the lower and upper confidence limits (CL) shown in parentheses. nd–no CL defined. (PDF) [file pone.0214702.s004.pdf]

S4 Table. Probit regression model parameters and fitted doses of active substances of insecticides describing the mortality of CSFB from Prague and Potěhy localities in 2018. The model was y = ax+b, with a being log transformed, and the lower and upper confidence limits (CL) shown in parentheses. nd – no CL defined.

**population**	**active substance**	**N**	**R^2**	***chi*-square**	**LC50 (95% CL)**	**slope +/- SE**
Prague	*lambda*-cyhalotrin	89	0.88	40.7	0.001 (0.0005/0.003)	1.49 ± 0.23
	tau-fluvalinate	91	0.51	30.2	0.13 (0.09/0.20)	1.99 ± 0.36
	chlorpyrifos	56	1.00	0.0004	0.14 (nd)	8.58 ± 436
	Biscaya	115	0.12	9.81	1.48 (0.54/33.7)	0.60 ± 0.19
Potěhy	*lambda*-cyhalotrin	98	0.71	33.6	0.004 (0.001/0.007)	1.23 ± 0.21
	tau-fluvalinate	85	0.47	28.6	0.08 (0.05/0.12)	1.73 ± 0.32
	Biscaya	121	0.02	1.33	3.47 (nd)	0.20 ± 0.18
